# Alcohol and the Adolescent Brain: Human Studies

**Published:** 2004

**Authors:** Susan F. Tapert, Lisa Caldwell, Christina Burke

**Affiliations:** Susan F. Tapert, Ph.D., is an associate professor in the Department of Psychiatry at the University of California, and program director of the Substance Abuse Mental Illness program, VA San Diego Healthcare System, both positions in San Diego, California. Lisa Caldwell is a research assistant at the Veterans Medical Research Foundation, San Diego, California. Christina Burke, M.A., is a senior research assistant in the Department of Psychiatry at the University of California, San Diego, and at the Veterans Medical Research Foundation, San Diego, California

**Keywords:** young adult, adolescent, heavy drinking, alcohol use disorder, brain function, AODR (alcohol and other drug related) structural brain damage, AODR neuropsychological disorder, cognitive development, cognitive ability, cognitive and memory disorder, risk factors, sensitization, causes of AODU (alcohol and other drug use), family AODU history, gender differences, AOD use pattern, age of AODU onset, comorbidity

## Abstract

Many people begin to drink alcohol during adolescence and young adulthood. Alcohol consumption during this developmental period may have profound effects on brain structure and function. Heavy drinking has been shown to affect the neuropsychological performance (e.g., memory functions) of young people and may impair the growth and integrity of certain brain structures. Furthermore, alcohol consumption during adolescence may alter measures of brain functioning, such as blood flow in certain brain regions and electrical brain activities. Not all adolescents and young adults are equally sensitive to the effects of alcohol consumption, however. Moderating factors—such as family history of alcohol and other drug use disorders, gender, age at onset of drinking, drinking patterns, use of other drugs, and co-occurring psychiatric disorders—may influence the extent to which alcohol consumption interferes with an adolescent’s normal brain development and functioning.

Several decades of research have shown that chronic heavy drinking is associated with adverse effects on the central nervous system and have revealed some of the processes that give rise to these effects. Yet it remains unclear when in the course of a person’s “drinking career” these central nervous system changes may emerge. Recent research suggests that heavy drinking may already affect brain functioning in early adolescence, even in physically healthy youths. This issue is important and interesting for at least two reasons. First, the brain continues to develop throughout adolescence and into young adulthood, and insults to the brain during this period therefore could have an impact on long-term brain function. Consistent with this assumption, animal studies have demonstrated that alcohol exposure during adolescence and young adulthood can significantly interfere with an animal’s normal brain development and function (for a review, see the accompanying article by Hiller-Sturmhöfel and Swartzwelder). Second, young adulthood is a period when most people make critical educational, occupational, and social decisions, and impaired cognitive functioning at this time could substantially affect their futures.

Questions regarding alcohol’s influence on brain development and function during adolescence are especially pertinent because heavy drinking is quite common among young people. For example, in one survey, 36 percent of 19- to 28-year-olds reported having consumed five or more drinks in a row in the preceding 2 weeks (Johnston et al. 2003). Another survey determined that 7 percent of 18- to 25-year-olds meet the diagnostic criteria for alcohol dependence (Substance Abuse and Mental Health Services Administration [SAMHSA] 2003). Thus, a substantial number of adolescents and young adults could be at risk for alcohol-related impairment of brain development and brain function.

This article reviews research on the impact of alcohol on the brains of young adults. It discusses several areas of brain function and development that appear to be affected, describes the extent of the harm observed as well as the factors that appear to moderate alcohol’s effects, and identifies high-risk groups of youths who are most likely to incur alcohol-related brain impairment.

## Effects on Neuropsychological Performance

One aspect of brain functioning that is commonly studied in youths as well as older adults is neuropsychological performance,^1^ which includes memory function, attention, visuospatial skills, and executive functioning (e.g., planning, abstract reasoning, and goal-directed behavior). Several studies have suggested that heavy alcohol use in young people appears to be associated with potentially long-term deleterious effects on neuropsychological functioning.

Brown and colleagues (2000) studied 33 alcohol-dependent adolescents, ages 15 and 16, who were in treatment for dependence on alcohol but not on other drugs. These teens had used alcohol an average of 753 times in their lives. A control group (*N* = 24) who had no history of alcohol and other drug (AOD) problems were matched with the alcohol-dependent adolescents on age, gender, socioeconomic status, education, and family history of AOD problems. Both groups of participants were given neuropsychological tests after 3 weeks of abstinence. The investigators found that the alcohol-dependent adolescents performed worse on tests of verbal and nonverbal memory than the control adolescents. They also found that alcohol-dependent adolescents who reported a greater number of alcohol withdrawal symptoms demonstrated poorer visuospatial functioning.

These findings were supported by a long-term study of 115 adolescents with AOD use disorders^2^ (Tapert and Brown 1999). The participants, ages 13 to 18 (average age, 16) at the beginning of the study, were recruited after completing inpatient treatment for AOD use disorders and were initially followed for 4 years. At the 4-year followup, the participants were divided into three groups based on their AOD use at followup:

- Nonabusers (*N =* 32) who had experienced no further AOD-related problems after the initial treatment.
- Abstinent abusers (*N* = 38) who had resumed harmful AOD use after treatment but had been abstinent for the 28 days prior to followup (or longer).
- Active abusers (*N* = 45) who had resumed harmful AOD use after treatment and had used AODs in the 28 days prior to followup.

The three groups were similar with respect to demographic characteristics (e.g., age, ethnicity, and family history of AOD problems), except that the active abuser group included more males.

In addition to drinking and drug use behavior, the investigators assessed the number of different withdrawal symptoms reported by the participants at the 4-year followup. Abstinent abusers (i.e., those who had been abstinent for the 28 days prior to assessment) reported an average of 1.4 different withdrawal symptoms, compared with an average of 3.1 different withdrawal symptoms reported by active abusers.

Directly after recruitment into the study, the groups had shown no significant differences on any of the neuropsychological measures assessed. At the 4-year followup, however, the abstinent and active abusers performed worse than the nonabusers on attention tasks, with the active abusers exhibiting the worst performance. In addition, participants who reported more withdrawal symptoms during the previous 3 months showed poorer visuospatial abilities, even when gender, history of head injury, history of a learning disability, number of grades completed, and socioeconomic status were considered. This latter observation suggests that the number of different alcohol withdrawal symptoms may be an important indicator of the degree of later alcohol-related cognitive impairment, at least among AOD-dependent young adults. Alcohol withdrawal symptoms were equally predictive of neuropsychological problems in alcohol-dependent as well as other-drug-dependent youths (although almost all drug-dependent youths were also alcohol dependent).

A continuation of this study evaluated participants 8 years after initial recruitment into the study (Tapert et al. 2002*a*). These analyses confirmed that lifetime history of alcohol withdrawal symptoms predicted cognitive performance, especially on visuospatial tests—that is, young people who had experienced a greater number of withdrawal symptoms performed worse on visuospatial tasks than did young people who had had fewer withdrawal symptoms.

Although these studies clearly have demonstrated that heavy alcohol use during adolescence and young adulthood is associated with poorer neuropsychological functioning, these impairments in young adults with alcohol use disorders (AUDs) (i.e., alcohol abuse and dependence) appear to be generally mild. For example, Eckardt and colleagues (1995) assessed neuropsychological functioning in 101 males, ages 18 to 35, with AUDs. This study found that almost all participants performed within the average range across tests. Nevertheless, young people who had consumed more alcohol throughout their lives performed more poorly, and young people who had abstained from alcohol for a longer time performed better. The investigators also noted that the study participants had experienced alcohol-related problems only for relatively short periods of time, which may contribute to the relatively mild impairment seen. But even if neuropsychological impairments generally are not severe, they represent a serious public health concern, considering how prevalent heavy drinking and alcohol-related problems are in young adults.

## Effects on Brain Structure

In addition to neuropsychological analyses, scientists have conducted imaging studies in teens and young adults with AUDs to determine the effects of heavy drinking on the changing brain structure during this developmental stage. These studies have revealed a variety of structural brain abnormalities associated with alcohol use.

In one study, De Bellis and colleagues (2000) used an imaging technique called magnetic resonance imaging (MRI) to investigate the sizes (i.e., volumes) of various brain structures in adolescents and young adults with and without AUDs (see the figure). These researchers measured the following:

- *Intracranial volume:* Volume enclosed by the skull.
- *Cerebral volume:* Volume of the largest and uppermost section of the brain, the cerebrum, which is divided into two halves or hemispheres.
- *Cortical gray-matter volume:* Volume of the gray mass that consists primarily of nerve cell bodies and forms the outer layer (i.e., cortex) of the cerebral hemispheres.
- *White-matter volume:* Volume of the white mass that makes up most of the cerebrum and consists primarily of nerve cell extensions (axons) through which nerve cells connect with each other.
- *Corpus callosum volume:* Volume of the bundle of nerve cell extensions that connect the cerebral hemispheres.
- *Amygdaloid volume:* Volume of the amygdala, an almond-shaped gray-matter mass located deep in each cerebral hemisphere, which may play an important role in the development of repeated AOD use and AOD dependence.
- *Hippocampal volume:* Volume of the hippocampus, a curved brain structure located in each cerebral hemisphere, which is critical to learning new information and forming memories.

**Figure:**
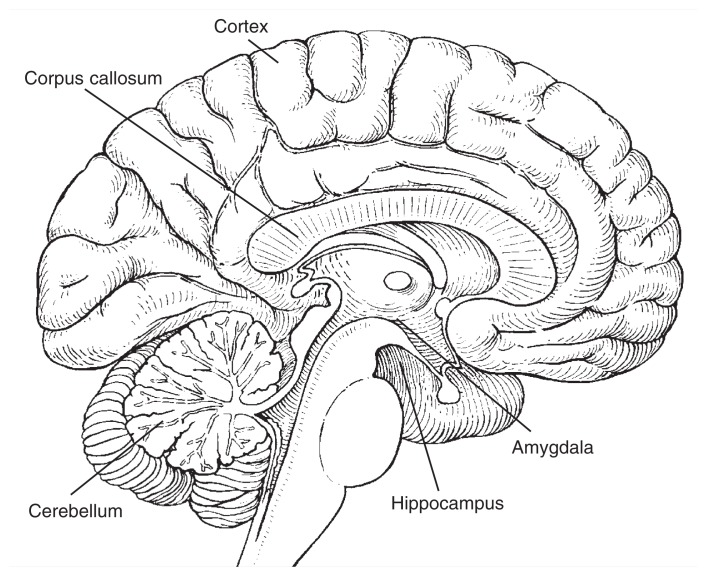
Areas of the brain that may be particularly vulnerable to alcohol’s effects. For example, the hippocampus, which lies deep within the cerebral hemispheres, plays an important role in learning and memory formation. Heavy drinking during adolescence can lead to reduced hippocampal volume.

When the investigators compared the volumes of these brain structures in adolescents and young adults with and without AUDs, only the hippocampal volumes differed significantly between the two groups. That is, left and right hippocampal volumes were significantly smaller in youths with AUDs than in matched control participants without AUDs. The researchers also found that hippocampal volume correlated positively with the age of onset of the AUD: The earlier a person developed an AUD, the smaller his or her hippocampi. Finally, a negative correlation was found between hippocampal volume and the duration of the AUD: Smaller hippocampi were found in participants who had longer-lasting AUDs than in subjects with AUDs of shorter duration. In another study of adolescents with and without AUDs who had no coexisting other drug use and other psychiatric disorders, Nagel and colleagues (in press) also detected smaller left hippocampal volumes in the adolescents with AUDs. These findings suggest that more enduring heavy-drinking patterns in adolescents and young adults are linked to smaller hippocampi and, because these brain structures are critical to learning and memory formation, may lead to more severe impairment of memory function.

Other studies that have looked at the effect of alcohol on the structure of white matter have identified subtle differences between youths with and without AUDs. In one preliminary study, Tapert and colleagues (2003) used a type of MRI technique, diffusion tensor imaging, to study the integrity of the white matter in the corpus callosum. This study determined that in youths with AUDs, white-matter integrity was reduced in the portion of the corpus callosum that is located toward the back of the brain (i.e., the splenium). Moreover, in these young people, white-matter integrity tended to be reduced in the rest of the corpus callosum as well, although the reductions were not statistically significant. The study also found that decreased white-matter integrity was significantly related to longer duration of heavy alcohol use, greater number of past alcohol withdrawal symptoms, and recent consumption of large amounts of alcohol.

Taken together, these imaging studies indicate that youths with AUDs may have some subtle abnormalities in hippocampal volume and white-matter integrity compared with demographically similar young people without AUDs. Even these subtle alterations could lead to disturbances in brain function that may have a long-lasting influence on subsequent performance of thinking and memory tasks. Similar impairment of hippocampal function and memory function resulting from alcohol exposure during adolescence has been demonstrated in animal models, further supporting the critical role of alcohol’s detrimental effects on this brain structure (for more information, see the accompanying article by Hiller-Sturmhöfel and Swartzwelder).

Although it is clear that heavy alcohol use can lead to changes in brain structure which can affect brain functioning, the reverse process—that an abnormality existing prior to alcohol exposure may predispose a person to alcohol use and the development of an AUD—also is plausible. In fact, both processes could occur in the same person, with a preexisting brain abnormality promoting heavy alcohol use, which then could lead to additional alterations in brain structure. To date, it is not known whether either of these processes takes precedence over the other or to what extent the processes co-occur in one person. This issue could be critically important to understanding the development of alcohol dependence in adolescents and young adults and to devising methods to prevent dependence, and therefore should be examined in future research that follows high-risk youths over time.

## Effects on Physiologic Brain Functioning

The term “brain functioning” refers to measures of the brain’s response (i.e., chemical and electrical processes) to thinking and memory tasks. Researchers have attempted to determine whether and how heavy alcohol use by adolescents and young adults relates to abnormalities in brain functioning. These approaches have used such techniques as measuring event-related potentials (ERPs) and performing functional MRIs (fMRIs).

Nichols and Martin (1993) examined physiological brain functioning in young adult heavy drinkers by measuring ERPs—brain waves that occur in response to a sudden stimulus or unexpected event. In particular, the researchers studied a brain wave called P300, which typically occurs 300 milliseconds after a stimulus and serves as a measure of information processing. In this study, participants looking at a series of pictures depicting a common event (in this case, safe driving) interspersed with pictures of a rare or “oddball” event (e.g., an accident scene) had to respond when the oddball event occurred. Participants performed the task both under the influence of a sedative drug (benzodiazepine) and after taking an inactive substance (i.e., placebo). The analyses revealed that under both conditions the P300 wave took longer to appear (i.e., had greater latency) in heavy-drinking young adults than in demographically comparable light drinkers. This result suggests that some information processing deficits exist that are related to heavy drinking and could represent an early stage along a continuum of alcohol-related brain damage.

The fMRI technique determines the activity in a brain region by detecting the amount of oxygen in the blood, which indicates the extent to which the brain region receives the oxygen it needs for nerve cell activity. If an fMRI is performed while the subject is engaged in a mental task, the level of activity in the brain regions involved can be identified. Tapert and colleagues (2004) used this approach to investigate brain response during a spatial working memory task in 15 adolescents with AUDs and 19 adolescents without AUDs (all ages 15 to 17). The participants were shown different abstract figures appearing one at a time in different locations on the screen, and were asked to press a button when a figure appeared in a repeat location. Youths with AUDs performed well on the task but showed greater blood oxygen levels in parietal regions and less response in some frontal and cerebellar areas compared with youths without AUDs. In a study of 10 alcohol-dependent young women ages 18 to 25 with longer histories of AUDs and 10 matched control women, however, the investigators found lower blood oxygen levels in many regions of the cerebral cortex in the alcohol-dependent women than in the control women (Tapert et al. 2001). This decreased response, which signifies reduced brain activity, corresponded with poorer performance on the task, particularly as the task progressed. This research indicates that these key brain regions may not receive adequate oxygenated blood for sustained accurate performance on the task. This reduced activity may represent a subtle neuronal disruption in these regions.

### Sensitivity to Alcohol

Numerous studies have described the effects of alcohol on the young brain, particularly with respect to sensitization (Chambers et al. 2003; Weissenborn and Duka 2003). The term “sensitization” refers to the fact that with increasing alcohol exposure, people may experience an intensified positive response to alcohol—that is, the reinforcing effect of drinking becomes more powerful,^3^ and certain other behavioral responses (e.g., aggression or intake of more alcohol or other drugs) become amplified. Some researchers have hypothesized that young people may be more likely to experience sensitization (and therefore may be more likely to consume excessive amounts of alcohol and develop AUDs) because their brains are still maturing, and the changes associated with this maturation could enhance the sensitization process.

For example, Chambers and colleagues (2003) proposed several reasons why young people might be particularly vulnerable to AOD use disorders. First, the brain continues to mature during adolescence, and disrupting the maturation process may damage brain function in the long term. Second, adolescents are more likely than people at other developmental stages to engage in impulsive behaviors, including heavy drinking. Impulse control is mediated by the prefrontal cortex, a region in the front of the brain.^4^ Because the prefrontal cortex continues to develop during adolescence, teens have not yet gained their full ability to control impulsive behavior and are therefore more prone to poor judgment related to drinking. One of the effects of consuming alcohol is that the brain releases a signaling molecule (i.e., a neurotransmitter) called dopamine in a brain region known as the nucleus accumbens. Dopamine release activates the nucleus accumbens, which in turn stimulates the brain’s reward system and triggers the desire for further positive stimulation, resulting in more alcohol use. Eventually, alcohol-related dopamine release may result in changes in brain development that lead to sensitization. This process also occurs in older people, but may be accelerated when it occurs in conjunction with the normal developmental changes in adolescents.

## Summary of Alcohol’s Effects

The preceding sections have shown that chronic heavy drinking during adolescence and into young adulthood appears associated with detrimental effects on brain development, brain functioning, and neuropsychological performance. Furthermore, imaging studies indicate that alcohol-consuming youth exhibit abnormalities in some brain areas that are particularly sensitive to disruption, such as the hippocampus, and in the chemical and electrical processes that occur during brain activity (e.g., in blood flow and the appearance of ERPs). These observations suggest that alcohol exposure during adolescence and young adulthood can cause subtle yet consequential damage. However, longitudinal studies that follow participants over several years will be needed to confirm this hypothesis.

## Moderating Factors

Although heavy drinking during adolescence can impair brain functioning, not all young people who drink heavily or become alcohol dependent will experience the same level of impairment, and some may show no damage at all. This variability in the effects of heavy alcohol consumption is attributable at least in part to a large number of moderating factors, such as genetic influences (as represented by the adolescent’s family history of AUDs), gender, age at onset of an AUD, drinking patterns and duration of abstinence, use of other drugs, and co-occurrence of other psychiatric disorders.

### Family History of AUDs

AUDs, like other psychiatric disorders, tend to run in families, indicating that genetic factors play a role in the development of these disorders. In fact, much alcohol research focuses on identifying the gene or genes that predispose a person to developing an AUD. It is plausible that predisposing genetic factors as evidenced by a family history of AUDs also influence the effects of alcohol consumption on the adolescent brain. To explore this relationship further, Tapert and Brown (2000) investigated the moderating effects of family history of AUD on neuropsychological functioning in adolescents ages 13 to 18. Study participants were classified in four groups:

- Detoxified AOD-dependent adolescents with a family history of AUDs.
- Detoxified AOD-dependent adolescents without a family history of AUDs.
- Non-AOD-abusing adolescents with a family history of AUDs.
- Non-AOD-abusing adolescents without a family history of AUDs.

The investigators administered a battery of neuropsychological tests of language, visuospatial ability, verbal memory, attention, and executive functioning to all four groups. They found that both AOD dependence and family history were associated with level of language and attention functioning. Specifically, the investigators made the following observations:

- Among adolescents who did not abuse AODs, those with a family history of AUDs had lower scores on language tasks than did those without such a family history, suggesting that genetic factors, or possibly family environment, may contribute to lower language skills in some adolescents.
- Among adolescents without a family history of alcohol use disorders, those who were AOD dependent performed worse on attention tasks than did those who were not AOD dependent, suggesting that heavy AOD use may interfere with attention skills.
- Performance levels on language and attention tasks were similar for AOD-dependent adolescents without a family history and for those who did not abuse AODs but had a family history of alcohol use disorders.

Hill and Shen (2002) also have assessed the influence of family history using ERP studies. These investigators found that children of alcoholics may be somewhat delayed in developing normal P300 patterns, yet appear to “catch up” to the levels seen in control subjects by young adulthood.

Taken together, these findings suggest that AOD dependence and family history of AUDs are distinct risk factors for decrements in neuropsychological performance, and these factors appear to impact different areas of neuropsychological functioning.

### Gender Differences

Research with older adults has repeatedly suggested that women are more vulnerable to some effects of alcohol. For example, women appear to develop liver damage after lower overall alcohol consumption than men (Department of Health and Human Services 1997). Women also are more vulnerable to alcohol’s toxic effects on the brain (Hommer et al. 2001; Schweinsburg et al. 2003). However, only a few studies have analyzed gender differences among young adults with AUDs. One study compared male and female adolescents with AUDs to nonabusing male and female control subjects on various neuropsychological tasks (Moss et al. 1994). This study found that alcohol-abusing males outperformed male control subjects on a problem-solving task, whereas females with AUDs performed worse than the female control subjects. The investigators postulated that alcohol may affect frontal lobe functioning, which is required for these problem-solving tasks, more readily in females than in males.

### Age at Onset of AUD

Because the brain continues to mature throughout adolescence, it is reasonable to speculate that the effects of heavy drinking on the brain may differ depending on the age at which an adolescent developed an AUD. A neuropsychological study investigating cognitive deficits as a function of age at onset and duration of an AUD found that participants with early-onset alcoholism (i.e., before age 35) showed the greatest degree of cognitive impairment (Pishkin et al. 1985).

Other researchers have examined the effects of age at onset of alcoholism using imaging techniques. Demir and colleagues (2002) used single photon emission computed tomography (SPECT) to measure blood flow through various brain regions. This regional cerebral blood flow (rCBF) is a measure of brain activity in those regions. Participants in this study were male alcoholics with early-onset (before age 20) and late-onset (after age 20) alcoholism as well as nonalcoholic control subjects. The investigators found that both early-onset and late-onset alcoholics showed impaired neuropsychological functioning and abnormal rCBF when compared with control subjects. Both groups of alcoholics had decreased rCBF in the left superior frontal regions compared with control participants. Late-onset alcoholics also showed decreased blood flow in the right superior frontal region. However, early-onset and late-onset alcoholics did not differ on most measures of rCBF and neuropsychological performance. In contrast to the study by Pishkin and colleagues (1985), these findings suggest that earlier onset of heavy drinking is not necessarily more detrimental to brain functioning than late onset.

### Drinking Patterns

Numerous studies have demonstrated that the effects of alcohol depend not only on the amount of alcohol consumed but also on the pattern of consumption. In general, drinking moderate alcohol amounts (one or two glasses of alcohol) almost every day appears to be less harmful than consuming the same total amount (that is, 7 to 14 glasses) on just one or two occasions per week—a pattern known as binge drinking or heavy episodic drinking. Heavy episodic drinking, which often is associated with hangover or mild withdrawal symptoms, is particularly common in adolescents and young adults, among whom this drinking pattern appears to be related to cognitive impairment. For example, in their 8-year followup study of adolescents with AUDs (see the earlier section, “Effects on Neuropsychological Performance”), Tapert and colleagues (2002*a*) found that having more hangover or alcohol withdrawal symptoms (which indicates a pattern of heavy episodic drinking) predicted poorer visuospatial functioning in young adulthood. This relationship was observed even after controlling for visuospatial functioning at the beginning of the study, AOD use, and practice effects from the previous administration of the neuropsychological tests.

Other studies, both in humans and in animal models, also have associated heavy episodic drinking patterns, as opposed to daily drinking patterns, with detrimental effects on cognitive functioning. These analyses also found that adolescents or young adults who are heavy episodic drinkers may be more sensitive to alcohol’s harmful effects on neurocognition than those who drink less or in a more consistent pattern. Weissenborn and Duka (2003) studied 95 participants ages 18 to 34, categorizing them as nonepisodic drinkers or heavy episodic drinkers, which are defined as men who consume five drinks or more per occasion and women who consume four drinks or more. Half of the participants from each group were given alcohol (the equivalent of about four to five drinks), and the others received a placebo. When all participants were subsequently tested on memory acquisition, motor functioning, spatial working memory, pattern recognition, and spatial recognition tasks—in the presence and absence of alcohol—the heavy episodic drinkers performed significantly worse than the nonepisodic drinkers on the spatial-working-memory and pattern recognition tasks. These findings support the hypothesis that a pattern of heavy episodic drinking can be particularly harmful to cognitive functioning.

### Duration of Abstinence

Studies in adult alcoholics have shown that some brain function previously damaged by alcohol can be recovered after sustained abstinence (Mann et al. 1999). Studies in young people suggest that at least some negative neuropsychological effects of heavy drinking persist even among drinkers who remain abstinent for extended periods of time (Brown et al. 2000; Tapert and Brown 2000; Tapert et al. 2002*a*). Brown and colleagues (2000) found that adolescents with AUDs still appeared to have reduced neuropsychological functioning after a 3-week period of abstinence, ruling out protracted withdrawal as the cause of reduced scores. However, it remains largely unknown whether full functioning returns after longer periods (e.g., 6 months to 1 year or more) of abstinence. More research is needed to determine if these consequences can reverse and whether the adolescent brain recovers from the effects of heavy alcohol use more easily than the adult brain.

### Use of Other Drugs

In addition to the growing research on the neurocognitive effects^5^ of heavy alcohol use, researchers have evaluated how using more than one drug affects neurocognitive functioning in young people. Grant and colleagues (1978) suggested that neuropsychological deficits were most strongly related to the heavy use of four or more drugs in addition to heavy alcohol use. These investigators concluded that heavy use of alcohol in conjunction with other drugs exacerbates the neuropsychological effects of alcohol alone among young adults. Other studies have produced different results, however, and there is tremendous variability among adolescents with respect to patterns of combining different drugs, which greatly complicates conducting studies and comparing the results. Nevertheless, it appears that using drugs such as cocaine or methamphetamine, or abusing prescription drugs in conjunction with heavy drinking, is related to poorer neurocognitive functioning.

### Co-Occurring Psychiatric Disorders

Youths with AUDs are at increased risk of having other psychiatric disorders as well, such as conduct disorder, bipolar disorder, schizophrenia, and attention deficit hyperactivity disorder (Myers et al. 1998; Tomlinson et al. 2004). At the same time, young people with psychiatric symptoms and disorders are more likely to have AUDs than are youths without psychiatric problems (Tapert et al. 2002*b*). In particular, conduct disorder (which is characterized by inappropriate, defiant, aggressive, or antisocial behavior in children and adolescents) is related to an increased risk of developing AOD use disorders in adolescence.

Giancola and colleagues (2001) have suggested that antisocial behavior is one of the best predictors of AOD use disorders. In their study, these investigators divided 282 female adolescents, ages 14 to 18, into two groups: an AOD use disorder group (*N* = 188) and a control group (*N* = 94) without AOD use or other psychiatric disorders. The two groups were matched on age, socioeconomic status, and intelligence. All participants were given tests measuring constructive thinking (i.e., ability to think in a manner that solves everyday problems efficiently) and executive functioning (e.g., planning, abstract reasoning, cognitive flexibility, working memory, and regulation of goal-directed behavior). The study found that the AOD use disorder group exhibited significantly poorer constructive thinking and executive functioning than did the control group. Moreover, the AOD abusers also reported a significantly higher level of antisocial behavior. Statistical analyses suggested that antisocial behavior was responsible for 77 percent of the relationship between AOD use disorder and executive functioning and for 51 percent of the relationship between AOD use disorder and constructive thinking. These results indicate that antisocial behavior is an important variable to consider when evaluating neurocognitive functioning in adolescents with AOD use disorders.

## Conclusions

Recent evidence suggests that heavy drinking during adolescence and young adulthood is associated with poorer neurocognitive functioning during the young adult years, and particularly with impairment of attention and visuospatial skills. This is an important area of research because a substantial portion of the young adult population drink at potentially harmful levels. Moreover, young adulthood, especially the college years between the ages of 18 and 25, is an important period in life during which key decisions in educational, occupational, and social realms are made that can have lifelong ramifications. Therefore, solid information on the causes and consequences of alcohol use during this developmental period is needed in order to devise effective strategies to prevent alcohol-related neurocognitive impairments.

Brain imaging and studies of event-related potentials have demonstrated that heavy alcohol consumption during adolescence and young adulthood also can lead to subtle but significant abnormalities in brain structure and function. The alterations observed include reduced hippocampal volume, disturbed white-matter integrity, delayed neural response during information processing, and reduced brain response in key regions during tasks requiring working memory. These abnormalities may represent subtle early harm to brain cells and other constituents that may result from the neurotoxic effects of alcohol.

Several factors influence the likelihood that a young adult drinker would incur these adverse neurocognitive effects. For example, a drinking pattern characterized by heavy and episodic drinking to the extent that the drinker experiences unpleasant effects (i.e., hangover or withdrawal symptoms) after drinking has been associated with particularly poor cognitive functioning. Being female and having a family history of alcoholism appear to be moderately associated with additional vulnerability to the deleterious effects of heavy drinking on neurocognition, but more research is needed in order to understand these effects in young people. In addition, it is essential to consider concurrent use of other drugs and the presence of other psychiatric disorders (e.g., conduct disorder) when assessing the relationships between alcohol consumption and brain functioning. Additional longitudinal studies will lead to a better understanding of how neurocognition is affected by age at onset of an AUD, the extent to which alcohol use actually causes brain problems, and the recoverability of brain functioning with sustained abstinence. Importantly, although there is much evidence that it is indeed alcohol (rather than preexisting genetic, brain, or psychiatric factors; brain abnormalities; or psychiatric disorders) that causes the adverse neurocognitive effects observed in the studies discussed in this article, this assumption has yet to be confirmed unequivocally.

Even though the research on alcohol’s effects in adolescence and young adulthood is still in its early stages, one message is clear: Young people can help maximize their neurocognitive potential by refraining from heavy drinking.

## Footnotes

1 The term “neuropsychological performance” refers to performance on standardized tests of thinking and memory skills.

2 All participants met the diagnostic criteria established in the *Diagnostic and Statistical Manual of Mental Disorders, Third Edition, Revised* (DSM–III–R) (American Psychiatric Association [APA] 1987) for abuse of or dependence on alcohol and at least one other drug.

3 “Reinforcing effects” are the consequences of alcohol use, such as feelings of well-being or euphoria, that promote further alcohol consumption.

4 The prefrontal cortex often appears to function abnormally in people with AOD use disorders.

5 The term “neurocognitive” refers to thinking and memory abilities as determined by a variety of measures, including brain imaging, nonstandardized thinking and memory tests, and neuropsychological tests.
